# Histopathological and Functional Evaluation of Radiation-Induced Sciatic Nerve Damage: Melatonin as Radioprotector

**DOI:** 10.3390/medicina55080502

**Published:** 2019-08-19

**Authors:** Dheyauldeen Shabeeb, Ahmed Eleojo Musa, Mansoor Keshavarz, Farid Esmaely, Gholamreza Hassanzadeh, Alireza Shirazi, Masoud Najafi

**Affiliations:** 1 Department of Medical Physics, School of Medicine, Tehran University of Medical Sciences, Tehran 1416753955, Iran; 2 Department of Physiology, College of Medicine, University of Misan, Misan 62010, Iraq; 3 Misan Radiotherapy Center, Misan Health Directorate, Ministry of Health/Environment, Misan 62010, Iraq; 4 Research Center for Molecular and Cellular Imaging, Tehran University of Medical Sciences, Tehran 1416753955, Iran; 5 Department of Physiology, School of Medicine, Tehran University of Medical Sciences, Tehran 1416753955, Iran; 6 Department of Anatomy, School of Medicine, Tehran University of Medical Sciences, Tehran 1416753955, Iran; 7 Radiology and Nuclear Medicine Department, School of Paramedical Sciences, Kermanshah University of Medical Sciences, Kermanshah 6715847141, Iran

**Keywords:** radiation, melatonin, neuropathy, peripheral nerve, sciatic nerve, radiotherapy

## Abstract

*Background and Objectives:* Radiotherapy uses ionizing radiation for cancer treatment. One of the side effects of radiotherapy is peripheral neuropathy. After irradiation, the first stage of neuropathy involves electrophysiological, biochemical and histopathological variations, while the fibrosis of soft tissues surrounding the exposed nerve occurs in the second stage. The present study aimed to examine the radioprotective effects of melatonin against ionizing radiation-induced sciatic nerve damage. *Materials and Methods:* Sixty male Wistar rats were assigned to four groups: C (Control + Vehicle), M (Melatonin), R (Radiation + Vehicle), MR (Radiation + Melatonin). Their right legs were irradiated with a 30 Gy single dose of gamma rays. Then, 100 mg/kg melatonin was administered to the animals 30 min before irradiation once daily (5 mg/kg) until the day of rats’ sacrifice. Their exposed nerve tissues were assessed using the sciatic functional index (SFI) and histological evaluation. *Results:* Four, 12 and 20 weeks post irradiation, the SFI results showed that irradiation led to partial loss of motor nerve function after 12 and 20 weeks. Histological evaluation showed the various stages of axonal degeneration and demyelination compared to the C and M groups. Scar-like tissues were detected around the irradiated nerves in the R group at 20 weeks, but were absent in the MR group. The SFI and histological results of the R group showed partial nerve lesion. However, in all cases, treatment with melatonin prevented these effects. *Conclusions:* Results showed that melatonin has the potential to improve functional and morphological features of exposed sciatic nerves. This could possibly improve the therapeutic window of radiotherapy.

## 1. Introduction

Radiotherapy is a treatment modality for cancer using ionizing radiation. It is administered to about 60% of cancer patients during the course of their treatment [1]. Despite its beneficial application, various side effects resulting from its use have been experienced. Major complications such as neurological damages, motor disability and pain, which reduce patients’ quality of life, have been reported [2]. Such effects result in permanent sensory and motor damages in the upper and lower limbs [2,3,4].

Radiation effects on tissues lead to the production of reactive oxygen species (ROS), reactive nitrogen species (RNS) as well as free radicals, which attack various cellular components such as DNA, lipids and proteins, causing cell death [5,6]. After irradiating the peripheral nerve, two stages of neuropathy arise. The first stage involves electrophysiological, biochemical and histopathological variations, while the fibrosis of soft tissues surrounding the exposed nerve occurs in the second stage [7]. These stages have key roles in the onset of peripheral neuropathy [3,8]. The degree of radiation-induced neuropathies depends on the location of the irradiated part, radiation dose and radiation delivery model [9]. The probability of neuropathy has been shown to increase with radiation dose [10]. Enzymes such as superoxide dismutase (SOD) and catalase (CAT) make up the antioxidant system. They protect cells against the side effects of free radicals. Radiation-induced damages to normal cells can give rise to an increase in the risk of cancer [11] as well as lipid peroxidation (LP) [12]. LP changes polyunsaturated fatty acids to malondialdehyde (MDA). MDA leads to cellular toxicity and a decrease in protective enzyme; it also acts as a co-carcinogenic agent [13]. SOD, the body’s major antioxidant defense which is mostly found in oxygen-based organisms, catalyzes the dismutation of O_2_^−^ to hydrogen peroxide (H_2_O_2_), thereby preventing further production of free radicals [14]. CAT, which is mostly found in small membrane-enclosed cellular components (peroxisomes), detoxifies H_2_O_2_ and other molecules through the catalysis of two H_2_O_2_ molecules to produce water (H_2_O) and oxygen (O_2_) [15].

Several pharmaceutical agents have been proposed for the mitigation of radiation-induced neuropathies. Melatonin (N-acetyl-5-methoxytryptamine) is a neurohormone produced by the pineal gland in a circadian manner, as well as in a non-circadian manner by other organs. It controls several physiological and pathological processes. In addition to its anti-inflammatory property, it protects tissues against radiation-induced injuries by scavenging free radicals [16,17]. In view of these properties, the present study aimed to examine the radioprotective effects of melatonin against ionizing radiation-induced sciatic nerve damage.

## 2. Methods

### 2.1. Animals

Sixty male Wistar rats (180–210 g) were purchased from Razi Institute in Tehran, Iran. They were kept in Plexiglas cages under a temperature of 21 °C, 50–70% relative humidity, an air flow rate of 15 exchanges per hour and a 12 h light and dark cycle.

### 2.2. Experimental Design

Full ethical approval for this study was obtained from the Ethical Committee of the School of Medicine, Tehran University of Medical Sciences (approval number 35116; 3 September 2017). We adhered to the recommendations of the Ethical Committee for the care and use of laboratory animals. All rats were distributed into four groups (15 rats in each) as follows: C (Control + Vehicle), M (Melatonin), R (Radiation + Vehicle), MR (Radiation + Melatonin).

Rats in the C group received only intraperitoneal injection of ethanol diluted with normal saline 0.9 NaCl. The final ethanol concentration was 5% with a vehicle period of 4 weeks. Intraperitoneal injection of melatonin dissolved in ethanol and normal saline was administered to the M group. The rats in the R and MR groups both received a single radiation dose of 30 Gy to their right legs [18,19]. However, only the MR group was administered with melatonin.

### 2.3. Irradiation

Rats were anesthetized using intraperitoneal injection of ketamine (90 mg/kg) and xylazine (10 mg/kg) before irradiation. Their right legs were exposed to a single dose of 30 Gy from a cobalt-60 gamma ray teletherapy unit at a source-to-skin distance (SSD) of 80 cm, field size of 10 × 20 cm^2^ and a dose rate of 0.65 Gy/min. Irradiation was done according to Öhrnel’s protocol, in which the single dose of 30 Gy in clinical practice would correspond to approximately 50–70 Gy applied in fractionated radiotherapy [20]. Other parts of the animals’ body were shielded using lead and beam collimation.

### 2.4. Drug Administration

First, 100 mg/kg of melatonin was dissolved in ethanol and diluted with sterile saline to give a final concentration of 5%. The intraperitoneal injection of this solution was administered 30 min before irradiation, while 5 mg/kg was given once daily for 4 weeks till the day of rats’ sacrifice [21,22,23]. These doses had no toxic effect [24,25,26,27].

### 2.5. Sciatic Nerve Function Index (SFI)

The sciatic function index (SFI) is one of the most commonly utilized functional evaluation methods. It uses mathematical methods to compare the parameters of normal footprints with experimental (affected foot). It also gives information on the recovery of motor–sensory connections as well as cortical integration related to the function of walking and mediated by the sciatic nerve [28]. Several improvements on the SFI have made it simpler and more reliable [29,30]. In the present study, prints predicted by a mathematical formula were conducted on an 8.2 × 42 cm^2^ corridor darkened at one end, while a white sheet of paper was placed on the floor. The paws of the rats were coated with black ink. Afterwards, they were allowed to walk around the corridor to obtain foot prints on the white paper. The calculation of their walking track analysis was given as follows:

Print length factor (PLF): [experimental print length (EPL) − normal print length (NPL)]/NPL. Toe spread factor (TSF): [experimental toe spread (ETS) − normal toe spread (NTS)]/NTS. Intermediary toe spread factor (ITF): [experimental intermediary toe spread (EIT) − normal intermediary toe spread (NIT)]/NIT.

**Figure 1:**
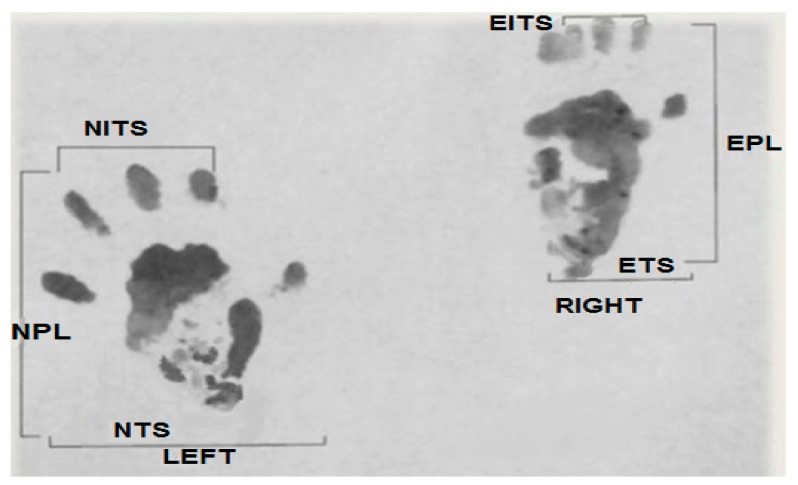
Original picture of a footprint from our study used to calculate parameters of the sciatic function index (SFI).

These parameters are presented in Figure 1.

Applying the Bain et al. equation, we obtain: SFI = −38.3 (PLF) + 109.5 (TSF) + 13.3 (ITF) − 8.8 [29]. Here, SFI = 0 represents normal function, while SFI = −100 represents complete functional loss.

### 2.6. Histopathological Examination

**Figure 2:**
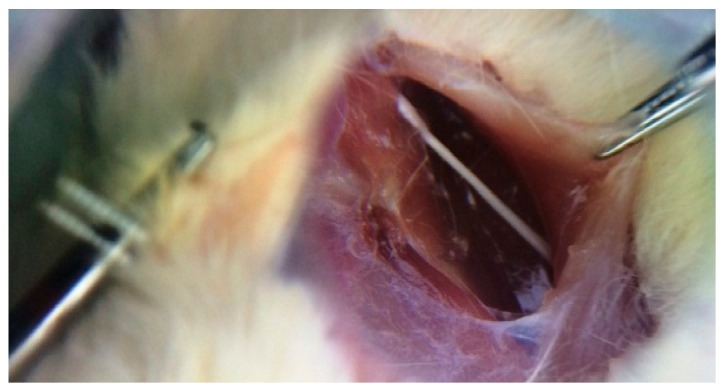
Sciatic nerve of the right leg (original picture from our study).

Five rats in each group were sacrificed 4, 12 and 20 weeks after irradiation. Their sciatic nerves were isolated from surrounding tissues by separating the femoral biceps and gluteal muscles using blunt dissection, as shown in Figure 2. The extracted nerves were fixed in 10% buffer formalin, dehydrated with an alcohol solution and embedded in paraffin. Five-millimeter transverse sections were obtained from the exposed sciatic nerves and stained with hematoxylin and eosin (H and E). Afterwards, the samples were assessed for histomorphological changes (degenerative, vascular and necrotic changes, as well as swellings in myelin sheath, axon, myelin sheath thickness and scar formation around the exposed nerve) with the aid of a light microscope. The semi-quantitative scoring of each variable was carried out by an experienced histopathologist and scored using the following scale: Grade 0 = within normal limits, Grade 1 = mild, Grade 2 = moderate, Grade 3 = severe injury [31].

### 2.7. Statistical Analysis

All statistical analyses were performed using SPSS software version 22 (IBM, Chicago, IL, USA). Data were presented as the mean ± standard deviation (SD). Differences between groups were analyzed using two-way ANOVA, in addition to Tukey’s multiple comparison tests. Mann-Whitney test was used for nonparametric histopathological comparisons at each time point. *p* value < 0.05 was considered statistically significant.

## 3. Results

### 3.1. Functional Assessment

**Figure 3:**
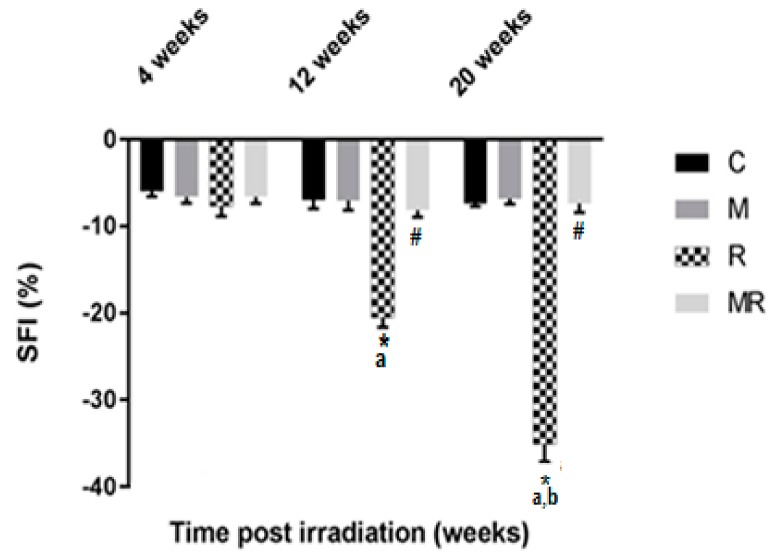
Effect of irradiation pre and post treatment with melatonin on the SFI changes at 4, 12 and 20 weeks post irradiation. * Significant difference from the C (Control) group (*p* < 0.001). # Significant difference from the R (Radiation) group (*p* < 0.001). The a indicates significant difference at 4 weeks (*p* <0.001). The b indicates significant difference at 12 weeks (*p* < 0.001).

Evaluation of the SFI showed that the R group had higher effects compared with the C and M groups after 12 and 20 weeks (*p* < 0.001). The average SFI values after 12 and 20 weeks were −20.51 ± 1.10 and −35.06 ± 2.00 in the R group, −6.98 ± 1.00 and −7.37 ± 0.35 in the C group as well as −7.06 ± 1.08 and −6.86 ± 0.58 in the M group, respectively. However, no significant effect was observed after 4 weeks (*p* > 0.05). After 12 and 20 weeks, the average SFI values were −8.13 ± 0.80 and −7.40 ± 1.01 in the MR group, respectively (*p* < 0.001). There was no significant difference between the SFI values of the C group compared with those of the MR group (*p* > 0.05). Only the R group was time-dependent (*p* < 0.001), as shown in Figure 3.

### 3.2. Histopathological Evaluation

**Figure 4:**
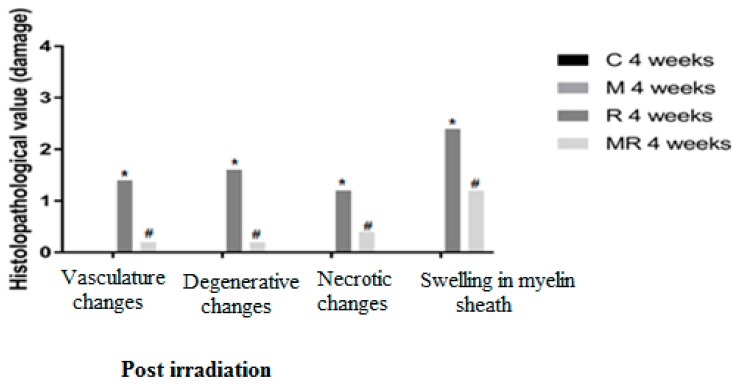
Effects of irradiation pre and post treatment with melatonin on histopathological changes at 4 weeks post irradiation. At different time periods, these variables for the control and melatonin groups were within normal limits equal to zero. * Significant difference from the C group (*p* < 0.05). # Significant difference from the R group (*p* < 0.01).

**Figure 5:**
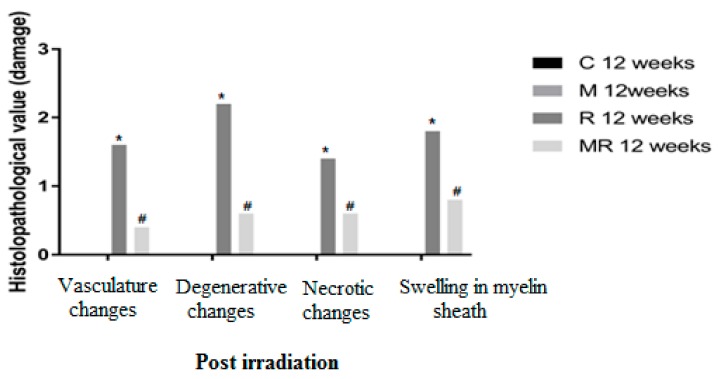
Effects of irradiation pre and post treatment with melatonin on histopathological changes at 12 weeks post irradiation. At different time periods, these variables for the control and melatonin groups were within normal limits equal to zero. * Significant difference from the C group (*p* < 0.05). # Significant difference from the R group (*p* < 0.01).

**Figure 6:**
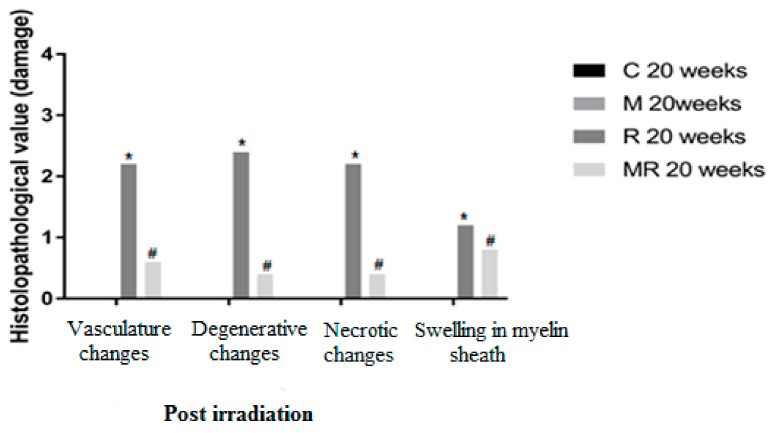
Effects of irradiation pre and post treatment with melatonin on histopathological changes at 20 weeks post irradiation. At different time periods, these variables for the control and melatonin groups were within normal limits equal to zero. * Significant difference from the C group (*p* < 0.05). # Significant difference from the R group (*p* < 0.01).

Four, 12 and 20 weeks post irradiation, the histological evaluation showed statistically significant differences between the R group and the C and M groups (*p* < 0.005) in terms of vasculature changes, degenerative changes, necrotic changes and swelling in myelin sheath. Scar-like tissues were detected significantly around the nerves in the R group compared with the C and M groups at 20 weeks post irradiation (*p* < 0.005), while treatment with melatonin prevented these effects in the MR group (*p* < 0.01). No significant finding was observed between the C and M groups and the MR group (*p* > 0.05), except for swelling at 4 weeks and degenerative changes, necrotic changes and swelling in myelin sheath at 12 weeks. There was time dependence in the R group (*p* < 0.05), hence suggesting melatonin as an efficient protective agent in all pathological conditions as shown in Figure 4, Figure 5 and Figure 6.

### 3.3. Axon and Myelin Sheath Diameter

**Figure 7:**
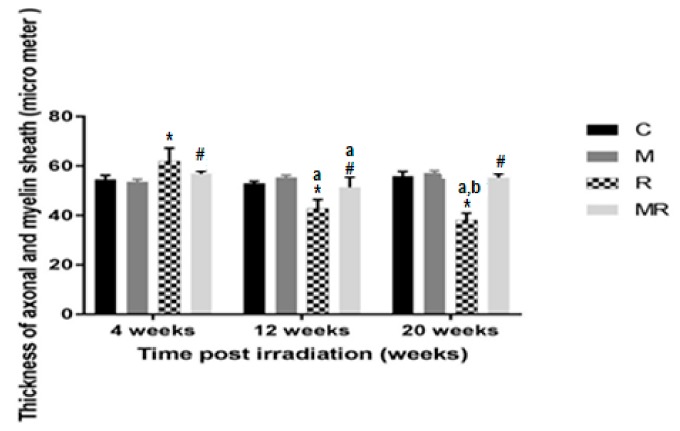
Effects of irradiation pre and post treatment with melatonin on the thickness of axonal and myelin sheath at 4, 12 and 20 weeks post irradiation. * Significant difference from the C group (*p* < 0.001). # Significant difference from the R group (*p* < 0.001). The a indicates significant difference at 4 weeks (*p* < 0.01). The b indicates significant difference at 12 weeks (*p* < 0.01).

**Figure 8:**
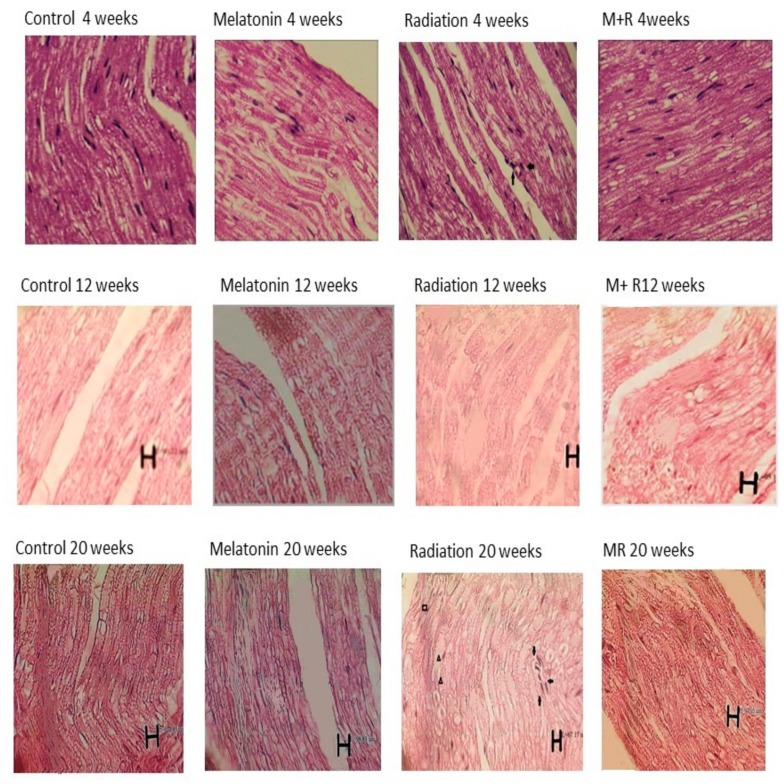
Histopathological changes (whose images are shown in longitudinal sections) at 4, 12 and 20 weeks post irradiation for all groups with 400× magnification and different scale bars. The control as well as melatonin groups showed appearance within normal limits. In the R group, we can see inflammatory cells in the sections and the aggregation of macrophages as well as the infiltration of polymorphonuclear and degenerative changes. In some nerve regions, the axon and myelin sheath diameters (measured using Image tool 2 of OPTIKA software, OPTIKA Microscope, Italy) were enlarged at 4 weeks due to inflammatory changes and congestion, while they were reduced at 12 and 20 weeks due to degenerative changes. For the MR (Melatonin + Radiation) group, we observed appearance within normal limits. Some artefacts which appeared in the histological pictures are not related to the structural anatomy of the nerve tissue. 🠪 Signifies inflammatory changes. ▲ Signifies degenerative changes and swelling in myelin sheath. ■ Signifies necrotic and vascular changes.

Four, 12 and 20 weeks post irradiation, the axon as well as myelin sheath diameters in the R group were significantly different compared with those in the C and M groups (*p* < 0.001). The average values of the C group were 54.61 ± 1.61 µm, 53.02 ± 0.83 µm and 55.95 ± 1.79 µm, respectively. In the M group, they were 53.64 ± 0.98 µm, 55.35 ± 0.94 µm and 57.09 ± 1.03 µm, respectively. In the R group, they were 62.09 ± 5.13 µm, 42.86 ± 3.59 µm and 38.18 ± 2.77 µm. Diameter measurements were significantly increased in the R group compared to the C and M groups at 4 weeks (*p* < 0.001), while they were significantly reduced in the R group compared to the C and M groups at 12 and 20 weeks (*p* < 0.001). Treatment with melatonin reversed these effects (*p* < 0.001). The mean values of the MR group were 56.83 ± 1.08 µm, 51.35 ± 4.13 µm and 55.30 ± 1.53 µm, respectively. There was no significant difference between the C and M groups (*p* > 0.05). Melatonin as well as radiation effects were both time-dependent (*p* < 0.01), except for in the MR group between 4 and 20 weeks (*p* > 0.05), as shown in Figure 7 and Figure 8.

## 4. Discussion

Radiotherapy is aimed at delivering the maximum radiation dose to kill tumor cells with minimal complications to normal tissues. However, normal tissues surrounding cancer cells are inevitably exposed to radiation, leading to early and late effects [32,33]. Peripheral neuropathy, one of the major side effects of radiotherapy, can lead to permanent sensory and motor damage in the upper and lower limbs [2,3,4,8,34,35]. Peripheral nerve (PN) abnormalities after exposure to ionizing radiation have been reported by several studies. Enzyme changes and increased glycogen uptake by PN have also been observed after exposure to a 30 Gy single radiation dose [36]. The direct effects of radiation on Schwann cells and radiation-induced fibrosis in the surrounding tissues of the nerves play a significant role in the onset of peripheral neuropathy [3,8]. Neuropathy after radiotherapy in breast cancer has also been reported [37,38,39]. The incidence of neuropathy has been observed in animal studies [40,41]. Scaravilli et al. showed that exposure to 20 Gy led to a reduction in nerve fibers as well as an increase in endoneurial collagen [42]. Other studies showed that irradiation led to a decline in nerve regeneration [43,44]. The risk of damages due to neuropathy is higher with increased total radiation dose and dose per fraction [45,46].

Radioprotectors can be used to reduce the risk of side effects after radiotherapy [47]. An appropriate radioprotector should protect healthy tissues without side effects due to the response of cancer cells [48,49]. Melatonin, a natural hormone in humans, has properties for protecting normal tissues against radiotherapy complications [50,51]. It can scavenge free radicals [52,53,54] and it has the advantages of less toxicity and minimal side effects [55,56].

Our results showed that irradiation led to partial loss of motor nerve function in terms of the SFI after 12 and 20 weeks. This finding agrees with previous studies which showed that the irradiation of peripheral nerves led to sensory and motor destruction [2,3,4,8,34,35]. In addition to the effect of a 30 Gy single radiation dose on SFI, there was also damage to the ankle joint, foot and toe movement. Therefore, the footprint typically showed increased print length, contracted toe spread and intermediate toe spread [57]. However, treatment with melatonin improved nerve function due to its direct free radical scavenging and indirect stimulation of the antioxidant enzyme. Melatonin offers superior treatment for peripheral nerve injury due to its special effects on the morphological features of the peripheral nerve tissue and positive effect on axon length, which lead to the sprouting of post peripheral nerve damages, inhibition of scar formation, improvement of nerve regeneration and functional recovery [58,59].

Histopathological examination is traditionally considered a descriptive approach. However, in recent years, several studies have shown that it is possible to conduct morphometric or quantitative analysis in histological sections [60]. Histological evaluation showed that the various stages of axonal degeneration and demyelination at 12 and 20 weeks were significantly induced by radiation compared to the measurements observed in the C and M groups. Free radical-induced LP has been proposed as a major factor of post nerve injury, which leads to the degeneration of nerve tissue [61,62]. Treatment with melatonin reversed these effects. Four weeks post irradiation, the axon and myelin sheath diameters of the R group increased due to inflammatory changes with moderate to severe swelling in myelin sheath, while the decrease in the diameter after 12 and 20 weeks was due to axonal degeneration and the demyelination of the sciatic nerve. The R group at 20 weeks showed scar-like formations around the exposed nerve, while treatment with melatonin inhibited these effects. Our results were close to those of a study by Gul et al. [63], which showed histological nerve variations such as myelin breakdown and axonal swelling or decline. Meanwhile, studies by Alimoradi et al. [64] and Pourmohammadi et al. [65] both showed histopathological changes in axonal degeneration and the demyelination of the sciatic nerve. Our histological evaluations are in agreement with the conclusions of these studies. Furthermore, in the present study, the SFI and histological results of the R group showed partial nerve lesion; however, melatonin was able to ameliorate these effects.

Radiation-induced neuropathy may be mostly unavoidable in radiotherapy for cancer patients. However, melatonin has the ability to prevent radiotherapy-induced complications via its anti-apoptotic and anti-inflammatory effects as well as its ability to regulate physiological and pathological processes [16,66]. It is more effective in scavenging free radicals, leading to lipid peroxidation [16]. In addition, studies have shown that melatonin can ameliorate chronic and acute inflammation [67]. It has ability to improve neurological defects by inhibiting lipid peroxidation and the synthesis of nitric oxide, decreasing oxidative mitochondrial damage, stimulating antioxidant enzymes and reducing edema as well as excitotoxic damages. Myelin sheath mainly consists of lipids. Its irradiation induces free radicals, which mediates lipid peroxidation. It has also been reported that long-term treatment with melatonin can ameliorate the oxidative damage of lipids, proteins as well as mitochondria in nerve tissue [68]. Melatonin is a more potent antioxidant than vitamins E and C as well as glutathione [52,53]. The hydroxyl radical induces lipid peroxidation and inhibits the synthesis of nitric oxide [69,70]. Hence, melatonin can improve the electrophysiological and functional features of irradiated sciatic nerves [71].

## 5. Conclusions

Results from this study have shown that melatonin has the potential to improve functional and morphological features of irradiated sciatic nerves. Hence, melatonin could be used to protect against radiotherapy-induced complications. Optimal and safe doses of melatonin should be administered over a long period of time for effective protection of the peripheral nerve tissues. This could go a long way in improving the therapeutic window of radiotherapy.
